# The PTPIP51 coiled-coil domain is important in VAPB binding, formation of ER-mitochondria contacts and IP3 receptor delivery of Ca^2+^ to mitochondria

**DOI:** 10.3389/fcell.2022.920947

**Published:** 2022-08-31

**Authors:** Gábor M. Mórotz, Sandra M. Martín-Guerrero, Andrea Markovinovic, Sebastien Paillusson, Matthew R. G. Russell, Pedro M. Pereira Machado, Roland A. Fleck, Wendy Noble, Christopher C.J. Miller

**Affiliations:** ^1^ Department of Basic and Clinical Neuroscience, Institute of Psychiatry, Psychology and Neuroscience, London, United Kingdom; ^2^ Centre for Ultrastructural Imaging, King’s College London, London, United Kingdom

**Keywords:** Alzheimer’s disease, Parkinson’s disease, amyotrofic lateral sclerosis, dementia, endoplasmic reticulum, mitochondria

## Abstract

Signaling between the endoplasmic reticulum (ER) and mitochondria regulates a number of fundamental physiological processes. This signaling involves close physical contacts between the two organelles that are mediated by the VAPB-PTPIP51 ″tethering” proteins. The VAPB-PTPIP51 tethers facilitate inositol 1,4,5-trisphosphate (IP3) receptor delivery of Ca^2+^ from ER to mitochondria. Damage to the tethers is seen in Alzheimer’s disease, Parkinson’s disease and frontotemporal dementia with related amyotrophic lateral sclerosis (FTD/ALS). Understanding the mechanisms that regulate the VAPB‐PTPIP51 interaction thus represents an important area of research. Recent studies suggest that an FFAT motif in PTPIP51 is key to its binding to VAPB but this work relies on *in vitro* studies with short peptides. Cellular studies to support this notion with full-length proteins are lacking. Here we address this issue. Immunoprecipitation assays from transfected cells revealed that deletion of the PTPIP51 FFAT motif has little effect on VAPB binding. However, mutation and deletion of a nearby coiled-coil domain markedly affect this binding. Using electron microscopy, we then show that deletion of the coiled-coil domain but not the FFAT motif abrogates the effect of PTPIP51 on ER-mitochondria contacts. Finally, we show that deletion of the coiled-coil domain but not the FFAT motif abrogates the effect of PTPIP51 on the IP3 receptor-mediated delivery of Ca^2+^ to mitochondria. Thus, the coiled-coil domain is essential for PTPIP51 ER-mitochondria signaling functions.

## Introduction

Signal transduction processes enable mammalian cells to communicate with each other and respond to physiological stimuli. An important component of this signaling involves communication between different organelles. Such crosstalk permits organelles to respond dynamically to changes in the cellular environment in an orchestrated manner (Cohen et al., 2018). Communications between the endoplasmic reticulum (ER) and mitochondria represent a particularly important component of organelle signaling since this regulates several fundamental cellular processes. These include bioenergetics, Ca^2+^ homeostasis, lipid metabolism, mitochondrial biogenesis and trafficking, ER stress responses, autophagy and inflammation (Paillusson et al., 2016; Csordas et al., 2018; Perrone et al., 2020; Markovinovic et al., 2022).

ER-mitochondria signaling is facilitated by close physical contacts between the two organelles such that up to 20% of the mitochondrial surface is tightly apposed to ER membranes. These regions of ER are termed mitochondria-associated ER membranes (MAM) (Paillusson et al., 2016; Csordas et al., 2018; Perrone et al., 2020; Markovinovic et al., 2022). The mechanisms by which ER membranes are recruited to the mitochondrial surface are not fully understood but it is widely accepted that the process involves “tethering proteins” that act to scaffold the two organelles in close proximity (Paillusson et al., 2016; Csordas et al., 2018; Perrone et al., 2020; Markovinovic et al., 2022). One of the best characterised tethers involves an interaction between the integral ER protein vesicle-associated membrane protein-associated protein B (VAPB) and the outer mitochondrial membrane protein, protein tyrosine phosphatase interacting protein-51 (PTPIP51) (De Vos et al., 2012; Stoica et al., 2014).

The VAPB-PTPIP51 tethers control a number of key cellular functions including IP3 receptor mediated delivery of Ca^2+^ from ER stores to mitochondria, bioenergetics, lipid synthesis, autophagy and, in neurons, synaptic activity (De Vos et al., 2012; Stoica et al., 2014; Galmes et al., 2016; Stoica et al., 2016; Gomez-Suaga et al., 2017; Paillusson et al., 2017; Gomez-Suaga et al., 2019; Yeo et al., 2021). Moreover, damage to the VAPB-PTPIP51 tethers and ER-mitochondria signaling is linked to the major human neurodegenerative diseases: Alzheimer’s disease, Parkinson’s disease and FTD/ALS (Stoica et al., 2014; Stoica et al., 2016; Paillusson et al., 2017; Lau et al., 2020; Gomez-Suaga et al., 2022). There is therefore much interest in the mechanisms that regulate the binding of VAPB to PTPIP51.

Recently, an FFAT (two phenylalanines in an acidic tract) motif in PTPIP51 was proposed as being key to its binding to VAPB (Di Mattia et al., 2020; Yeo et al., 2021). FFAT motifs are known to mediate interactions between cytoplasmic and ER proteins (Murphy and Levine, 2016). However, the above findings all involve *in vitro* binding studies with short PTPIP51 peptides spanning the FFAT motif (Di Mattia et al., 2020; Yeo et al., 2021). Complementary cellular data with full-length proteins are lacking. To address these issues, we used a number of cellular assays to probe the role of the FFAT motif in the binding of full-length PTPIP51 to VAPB, ER-mitochondria tethering and IP3 receptor mediated delivery of Ca^2+^ from ER stores to mitochondria which are primary functions of the VAPB-PTPIP51 tethers. We find that deletion of the FFAT motif has little effect on any of these functions. Rather, we identify a nearby coiled-coil domain in PTPIP51 that is essential for all of these functions.

## Materials and methods

### Plasmids

pCI-neo, EGFP-C1, and DsRed2-C1 control empty vectors were from Promega and Clontech; DsRed-Mito was from Clontech. Myc-tagged human VAPB and HA-tagged human PTPIP51 plasmids were as described previously (Stoica et al., 2014). Mutant PTPIP51 plasmids were generated using a Q5 site-directed mutagenesis kit (New England Biolabs) according to the manufacturer’s instructions. All mutant plasmids were verified by sequencing. PTPIP51-ΔFFAT-HA was created using oligonucleotides 5′-GGA​GCC​ACG​TTC​ACA​GAT​G-3′ and 5′-AGA​GCT​GGA​GCC​AGT​GGA-3'. PTPIP51-ΔC-C-HA was created using oligonucleotides 5′-CGA​GGG​CTT​GCG​GGG​GAG-3′ and 5′-CAC​CTT​CTC​CTG​TCC​TTC​CCG​T-3'. PTPIP51-C-Cmut-HA was created using oligonucleotides 5′-TGT​GGC​GCT​GCG​GCG​GGA​GCC​GGA​GGA​GCC​GAG​AAG​CAG​CCT​GCG​AGG​G-3′ and 5′-GGG​CTG​GTC​GGC​ACA​AAG​TCC​GGG​CGG​TCC​GGC​ACC​TTC​TCC​TGT​CCT​TCC​CGT​G-3'.

### Antibodies

Rabbit anti-hemagglutinin (HA) was obtained from Sigma. Mouse anti-Myc 9B11 and mouse anti-Ha 6E2 were from Cell Signaling. Antibodies were used at 1/2000 on immunoblots and 1/250 for immunoprecipitation and immunostaining.

### Cell culture and transfection

Human embryonic kidney-293 (HEK293) cells were grown in Dulbecco’s modified Eagle’s medium with 4.5 g/l glucose (Sigma) and SH-SY5Y cells were grown in Dulbecco’s modified Eagle’s medium/F12 (1:1) containing 3.15 g/l glucose (Thermo Scientific). Both media were supplemented with 10% (v/v) foetal bovine serum, 2 mM L-glutamine, 100 IU/ml penicillin, and 100 μg/ml streptomycin (Invitrogen). HEK293 cells were transfected with polyethylenimine MAX (Polysciences) and SH-SY5Y cells were transfected with Lipofectamine 2000 (Invitrogen) according to the manufacturer’s instructions. Cells were analysed 24 h post-transfection.

### Immunoprecipitation assays, SDS-PAGE and immunoblotting

Immunoprecipitation assays were performed essentially as described previously (Morotz et al., 2019). Briefly, cells were washed with phosphate buffered saline (PBS) and harvested by scraping into ice-cold immunoprecipitation lysis buffer (50 mM Tris-citrate pH 7.4, 150 mM NaCl, 1% (v/v) Triton X-100, 5 mM EGTA, 5 mM EDTA and protease inhibitors (Complete, Roche)) and incubating for 30 min. Following centrifugation at 15,000 x g for 15 min at 4°C, samples were incubated with primary antibody for 16 h at 4°C. Antibodies were captured using Protein G-sepharose beads (Sigma and Abcam; 50% (v/v) in PBS supplemented with 0.1% (v/v) Triton X-100) for 2 h at 4°C, washed in capture buffer and bound proteins eluted and prepared for SDS-PAGE by incubation in SDS-PAGE sample buffer and heating at 96°C for 5 min. Samples were separated on 10% gels using Mini-PROTEAN two gel electrophoresis systems (Bio-Rad) with a discontinuous buffer system. Separated proteins were transferred to BioTrace NT nitrocellulose membrane (0.2 μm pore size; Pall Corporation) using a Mini Trans-Blot electrophoretic transfer cell (Bio-Rad) for 1.5 h. Membranes were blocked with Tris-HCl buffered saline (TBS) containing 5% (w/v) milk powder and 0.1% (v/v) Tween-20 for 1 h. Membranes were probed with primary antibodies in blocking buffers supplemented with 0.1% (v/v) Tween-20 (TBS/Tween-20), washed in TBS/Tween-20 and incubated with IRDye-conjugated secondary antibodies in wash buffer and proteins visualised using an Odyssey CLx near infrared imaging system (Li-Cor Biosciences). Signals were quantified by Image Studio Lite (version 5.2.5; Li-Cor Biosciences).

### Electron microscopy and super resolution structured illumination microscopy

HEK293 cells were transfected with EGFP plus pCI-neo, EGFP plus PTPIP51-HA, EGFP plus PTPIP51-ΔFFAT-HA or EGFP plus PTPIP51-ΔC-C-HA and transfected cells isolated using a cell sorter with the EGFP channel essentially as described previously (Stoica et al., 2014). Immunostaining for the HA confirmed double transfection. Cells were plated onto 13 mm diameter glass coverslips (VWR International) and fixed with 2.5% glutaraldehyde in 0.1 M sodium cacodylate buffer pH 7.2 for 3 h at 20°C. Cells were then washed in 0.1 M sodium cacodylate buffer 3 × 5 min before and after staining for 1 h in 2% osmium tetroxide and 1.5% ferrocyanide in 0.1 M sodium cacodylate buffer at 4°C in the dark. The cells were then washed in water and stained with 1% uranyl acetate in water at 4°C in the dark for 1 h. After further washing cells were dehydrated in 70%, 90%, and twice in 100% ethanol and embedded in Spurr S024/D resin (TAAB). 150 nm sections were cut on a UC7 ultramicrotome (Leica) and stained for 6 min in Lead Citrate 22410 (Electron Microscopy Sciences, United States). Sections were imaged on a JEM-1400 FLASH transmission electron microscope using the Limitless Panorama program (JEOL); map montages were first acquired at 34.4 nm pixels (500X), then montages were acquired at 2.2 nm pixels (8000x) with deflector limit 5, margin 20%, and wait 0.5 s, and motor margin at 2 μm (11%), and wait 5 s. ER-mitochondria contacts were quantified by determining the proportion of the mitochondrial surface (expressed as a %) that was closely apposed (less than 30 nm) to ER and all clearly identified ER were quantified. This approach has been used in many studies e.g. (Csordas et al., 2006; Stoica et al., 2014; Stoica et al., 2016; Paillusson et al., 2017). In previous studies, we quantified these contacts manually but in the present study, we utilised a recently described ImageJ plugin; MitoCare Tools (Bartok et al., 2019). Using MitoCare Tools, mitochondrial circumferences and ER membranes facing toward mitochondria were first marked using the polygon and segmented line selection tools. MitoCare Tools was then used to measure the distance between mitochondria and ER on an orthogonal line for each point of the mitochondrial boundaries, and to then calculate mitochondrial circumference and binned interface length where the two organelles were closer than 30 nm.

For SIM analyses, cells were transfected with DsRed-Mito to label mitochondria and either HA-tagged wild-type PTPIP51 or PTPIP51 mutants in which the FFAT motif and coiled-coil domains were either deleted or mutated (wild-type PTPIP51-HA, PTPIP51-ΔFFAT-HA, PTPIP51-ΔC-C-HA and PTPIP51-C-Cmut-HA). Cells were fixed in 4% paraformaldehyde/0.1% glutaraldehyde and immunostained using rabbit anti-HA as described previously (Stoica et al., 2014). Goat anti-rabbit Igs coupled to AlexaFluor-647 were from Invitrogen. SIM was performed as described using a Nikon Eclipse Ti-E Inverted microscope with 100 × 1.49 NA CFI objective and equipped with Visitech iSIM Super Resolution System (Gomez-Suaga et al., 2019; Gomez-Suaga et al., 2022). Images were captured using an Andor iXon EMCCD camera and reconstructed using Nikon deconvolution software for iSIM.

### Calcium measurements

Mitochondrial and cytosolic Ca^2+^ levels were measured with Rhod-2/AM and Fluo4/AM respectively (Invitrogen) essentially as described previously (De Vos et al., 2012; Stoica et al., 2014; Stoica et al., 2016; Gomez-Suaga et al., 2017; Paillusson et al., 2017). Briefly, SH-SY5Y cells were plated on 18 mm diameter round coverslips (Marienfeld) and for mitochondrial Ca^2+^ measurements, transfected with EGFP and control empty vector or EGFP plus either PTPIP51-HA, PTPIP51-ΔFFAT-HA or PTPIP51-ΔC-C-HA. For cytosolic Ca^2+^ measurements, cells were transfected with DsRed plus either PTPIP51-HA, PTPIP51-ΔFFAT-HA or PTPIP51-ΔC-C-HA. Transfected cells were selected for analyses via EGFP (for mitochondrial Ca^2+^ measurements) or DsRed (for cytosolic Ca^2+^ measurements) signals. 24 h post transfection, cells were loaded with either 2 μM Rhod2/AM or 2 μM Fluo4/AM in external solution (145 mM NaCl, 2 mM KCl, 5 mM NaHCO_3_, 1 mM MgCl_2_, 2.5 mM CaCl_2_, 10 mM glucose, 10 mM Na-HEPES pH 7.25) in the presence of 0.02% (v/v) Pluronic-F27 (Invitrogen) for 15 min. After removing the dye, cells were washed with external solution for 15 min at 37°C, mounted in a Ludin imaging chamber (Life Imaging Systems, Basel, Switzerland) and kept under constant perfusion in external solution (0.5 ml/min) using an Ismatec REGLO peristaltic pump (IDEX Corporation, Glattbrugg, Switzerland). To invoke Ca^2+^ efflux from ER stores and measure changes in Ca^2+^ levels, 100 μM oxotremorine-M (Tocris) was applied in the external solution. In addition, Rhod2/AM mitochondrial Ca^2+^ levels were also monitored in Ca^2+^ free media (external solution lacking Ca^2+^ and including 500 μM EGTA). Rhod2/AM and Fluo4/AM images were recorded in time-lapse mode (2 s interval, 100 ms exposure) using a Nikon Eclipse Ti-2 microscope driven by NIS Elements AR software and equipped with Intenslight C-HGFI light source, CFI Plan Fluor 40x/1.4 NA objective, Andor Neo scientific complementary metal-oxide-semiconductor camera (Andor Technology). Filter sets were from Chroma Technology. Data were analysed with NIS Elements AR software. Mitochondrial and cytosolic Ca^2+^ levels were expressed as fluorescence signals after oxotremorine-M treatment relative to the average baseline before oxotremorine-M application (F/F0).

### Statistical analyses

Statistical analysis was performed using Excel (Microsoft Corporation) and Prism software (version 9.3.1; GraphPad Software Inc.). A detailed explanation of these analyses is described in the Figure legends.

## Results

### Deletion and mutation of the coiled-coil domain and FFAT motif do not affect mitochondrial localisation of PTPIP51

**FIGURE 1:**
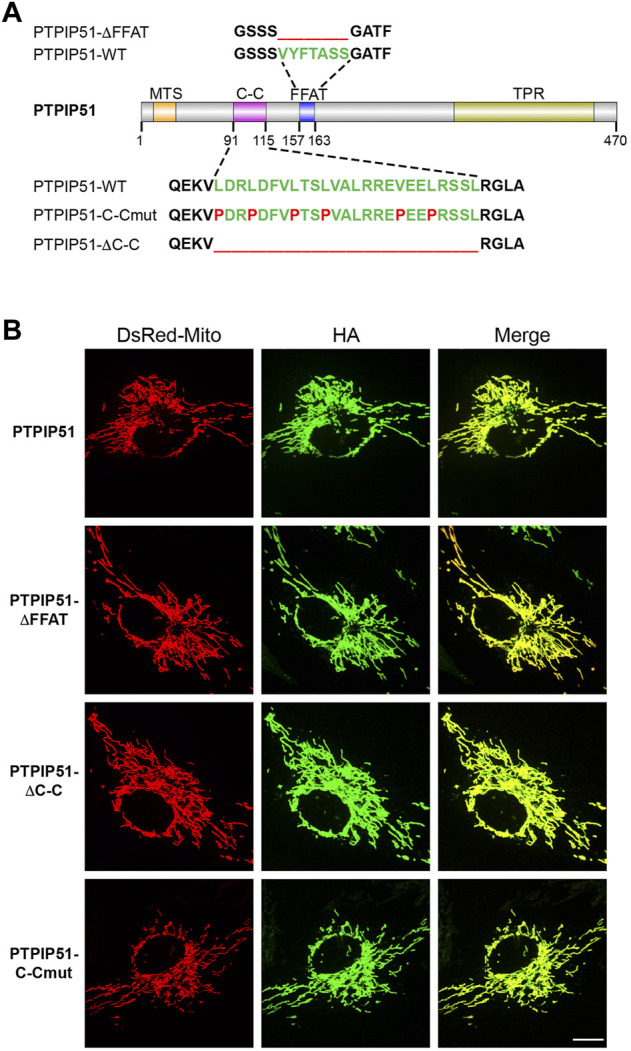
Disruption of the PTPIP51 coiled-coil domain and FFAT motif does not affect PTPIP51 mitochondrial localisation. **(A)** structure of PTPIP51 showing the N-terminal mitochondrial targeting sequence (MTS), coiled-coil domain (C–C), FFAT motif and C-terminal tetratricopeptide repeat domain (TPR). Also shown are mutant sequences in which the FFAT motif and coiled-coil domain were entirely deleted and in which disrupting prolines were introduced into the coiled-coil domain. **(B)** Mitochondrial localisation of the wild-type, FFAT and coiled-coil PTPIP51 mutants used in this study. Cells were transfected with DsRed-Mito and HA-tagged wild-type PTPIP51, PTPIP51-ΔFFAT, PTPIP51-ΔC-C, or PTPIP51-C-Cmut. Cells were immunostained for transfected PTPIP51 via the HA-tag and analysed by SIM. Scale bar = 10 μm.

PTPIP51 contains an N-terminal mitochondrial targeting sequence, a centrally located coiled-coil domain and FFAT motif, and a C-terminal tetratricopeptide repeat domain (Figure 1A). The smallest identified region of PTPIP51 that binds to VAPB involves amino-acids 75–174 which encompasses the coiled-coil domain and FFAT motif (De Vos et al., 2012). To probe the roles of the FFAT motif and coiled-coil domain in mediating binding to VAPB, ER-mitochondria contacts and IP3 receptor mediated delivery of Ca^2+^ to mitochondria, we created PTPIP51 mutants in which the FFAT motif and coiled-coil domain were disrupted. We made mutants in which the FFAT motif and coiled-coil domain were separately deleted (PTPIP51-ΔFFAT-HA and PTPIP51-ΔC-C-HA) and a further mutant in which disrupting prolines were introduced into the coiled-coil heptad repeats (PTPIP51-C-Cmut-HA) (Figure 1A). This is a well characterised and commonly used approach for disrupting coiled-coil domains and binding of their ligands as it introduces a kink into the coiled-coil structure (Chang et al., 1999; Fiumara et al., 2010).

We first confirmed that these PTPIP51 mutants all localise to mitochondria using super resolution iSIM. To do so, we transfected HEK293 cells with DsRed-Mito to label mitochondria and either HA-tagged wild-type PTPIP51 or the PTPIP51 coiled-coil or FFAT mutants and labelled transfected PTPIP51 via the HA tags. These experiments revealed that all PTPIP51 mutants were localised to mitochondria (Figure 1B). Such findings are consistent with previous studies which showed that deletion of the N-terminal mitochondrial targeting sequence abrogates mitochondrial localisation of PTPIP51 and fusion of this targeting sequence to EGFP drives EGFP to mitochondria (De Vos et al., 2012).

### Mutation and deletion of the PTPIP51 coiled-coil domain but not the FFAT motif markedly affects the binding of PTPIP51 to VAPB in cellular immunoprecipitation assays

**FIGURE 2:**
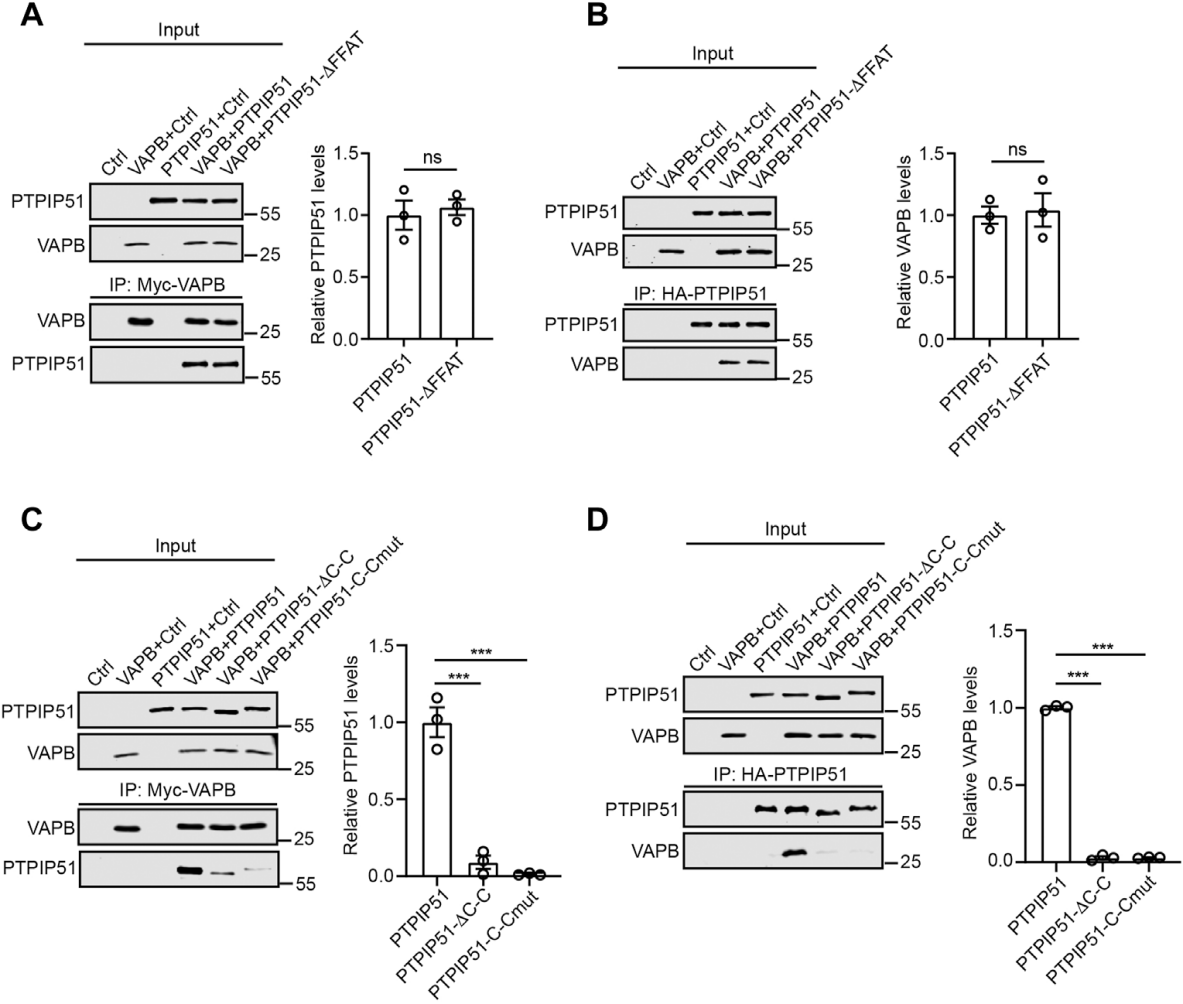
The PTPIP51 coiled-coil domain but not the FFAT motif is required for binding to VAPB in immunoprecipitation assays. **(A,B)**, deletion of the FFAT motif does not affect PTPIP51 binding to VAPB. HEK293 cells were transfected with control empty vector (Ctrl), myc-VAPB plus Ctrl, PTPIP51-HA plus Ctrl, myc-VAPB plus PTPIP51-HA or myc-VAPB plus PTPIP51-ΔFFAT-HA in which the FFAT motif was deleted. VAPB **(A)** or PTPIP51 **(B)** were immunoprecipitated using the myc- and HA-tags respectively and the amounts of bound PTPIP51-HA or myc-VAPB detected by immunoblotting using HA and myc antibodies. **(C,D)**, deletion and mutation of the PTPIP51 coiled-coil domain markedly disrupt binding to VAPB. Cells were transfected with control vector (Ctrl), myc-VAPB plus Ctrl, PTPIP51-HA plus Ctrl, myc-VAPB plus PTPIP51-HA, myc-VAPB plus PTPIP51-ΔC-C-HA or myc-VAPB plus PTPIP51-C-Cmut-HA. VAPB **(C)** or PTPIP51 **(D)** were immunoprecipitated using the myc- and HA-tags respectively and the amounts of bound PTPIP51-HA or myc-VAPB detected by immunoblotting using HA and myc antibodies. No signals were obtained for bound PTPIP51 **(A,C)** or VAPB **(B,D)** in immunoprecipitations from control-transfected cells or in cells transfected with myc-VAPB or PTPIP51-HA alone which demonstrates the specificity of the assays. Both inputs and immunoprecipitations (IP) are shown in A-D. Bar charts show the relative level of bound PTPIP51-HA or myc-VAPB in the assays. In A and B, data were analysed by unpaired t-test. In C and D, data were analysed by one-way analysis of variance (ANOVA) and Dunnett post-hoc test. N = 3 in all assays. Error bars are standard error of means (s.e.m.) ns, not significant, ***p ≤ 0.001.

To probe the roles of the FFAT motif and coiled-coil domain in mediating binding of full-length PTPIP51 to VAPB in cells, we performed immunoprecipitation assays from HEK293 cells transfected with control vector, myc-tagged VAPB (myc-VAPB), HA-tagged wild-type PTPIP51 or myc-VAPB plus either wild-type PTPIP51-HA or the PTPIP51 FFAT and coiled-coil domain mutants (PTPIP51-ΔFFAT-HA and PTPIP51-ΔC-C-HA, PTPIP51-C-Cmut-HA). We immunoprecipitated transfected myc-VAPB using the myc tag and probed for binding of co-transfected PTPIP51-HA on immunoblots; we also performed the reverse experiment in which PTPIP51-HA was immunoprecipitated using the HA tags and binding of myc-VAPB analysed. In these assays, deletion of the FFAT motif had little effect on the PTPIP51-VAPB interaction. However, deletion and mutation of the coiled-coil domain markedly disrupted binding (Figures 2A–D).

### Deletion of the PTPIP51 coiled-coil domain but not FFAT motif disrupts ER-mitochondria contacts

**FIGURE 3:**
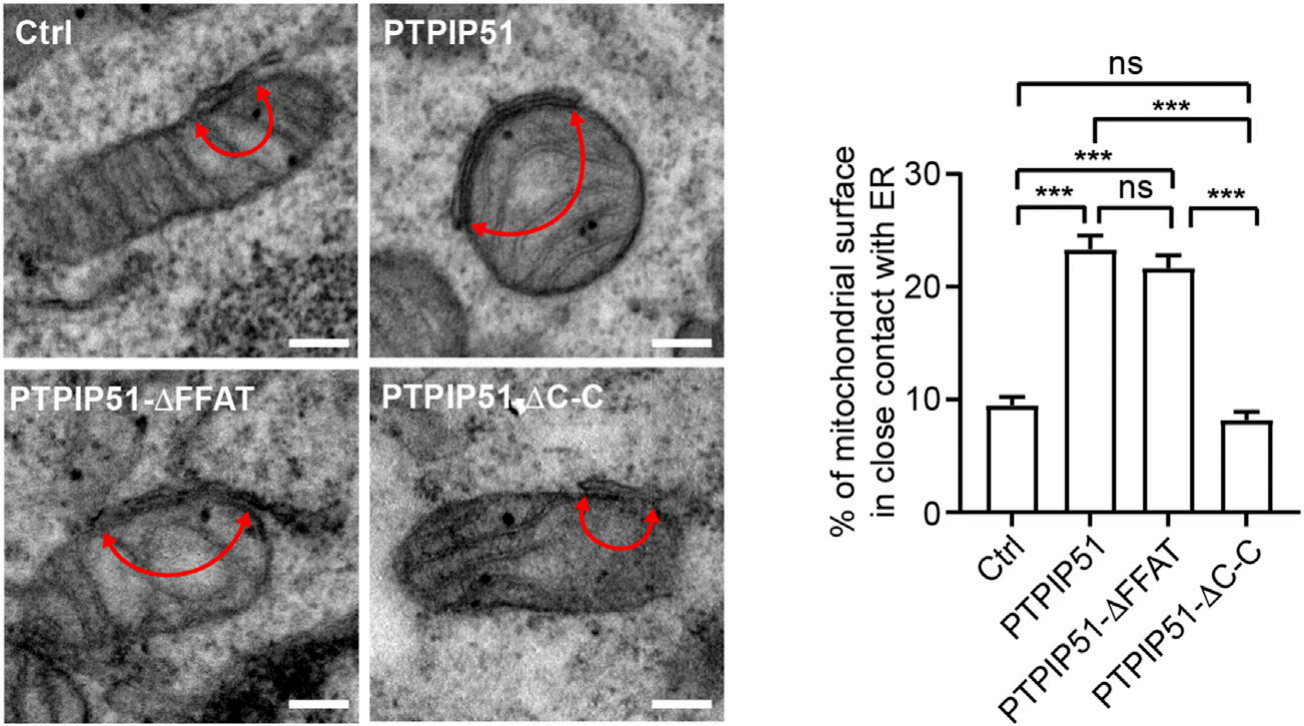
Deletion of the PTPIP51 coiled-coil but not FFAT motif disrupts ER-mitochondria contacts. HEK293 cells were transfected with EGFP plus either control vector, PTPIP51-HA, PTPIP51-ΔFFAT-HA or PTPIP51-ΔC-C-HA. Transfected cells were isolated using a cell sorter and the EGFP signal, replated and analysed by EM. Representative electron micrographs of ER–mitochondria contacts in control (Ctrl) PTPIP51-HA, PTPIP51-ΔFFAT-HA or PTPIP51-ΔC-C-HA transfected cells are shown. Arrowheads with loops show regions of contact. Scale bars = 200 nm. The bar chart shows the % of the mitochondrial surface in close (<30 nm distance) contact with ER. Data were analysed by Welch ANOVA and Games-Howell’s post hoc test. N = 20–32 cells and 98–124 mitochondria. Error bars are s. e.m. ns, not significant, ***p ≤ 0.001.

The binding of PTPIP51 to VAPB acts as a molecular scaffold that tethers regions of ER to mitochondria (Stoica et al., 2014). We therefore enquired how deletion of the PTPIP51 FFAT motif or coiled-coil domain affected ER-mitochondria contacts compared to wild-type PTPIP51. To do so, we transfected HEK293 cells with EGFP control vector or EGFP plus either wild-type PTPIP51 or PTPIP51 in which the FFAT motif and coiled-coil domains were separately deleted (PTPIP51-ΔFFAT or PTPIP51-ΔC-C) and used a cell sorter to isolate co-transfected cells for analyses by electron microscopy. Contacts were quantified by determining the proportion of the mitochondrial surface that was closely apposed (less than 30 nm) to ER. Such an approach has been used in many studies e.g. (Csordas et al., 2006; Stoica et al., 2014; Stoica et al., 2016; Paillusson et al., 2017). In agreement with previous studies, the expression of wild-type PTPIP51 significantly increased ER-mitochondria contacts (Stoica et al., 2014). Expression of PTPIP51-ΔFFAT also increased ER-mitochondria contacts to levels indistinguishable from wild-type PTPIP51. However, expression of PTPIP51-ΔC-C had no significant effect on ER-mitochondria contacts compared to control EGFP expressing cells (Figure 3).

### Deletion of the PTPIP51 coiled-coil domain but not FFAT motif disrupts IP3 receptor mediated delivery of Ca^2+^ to mitochondria

A primary physiological function of the VAPB-PTPIP51 tethers is to facilitate IP3 receptor mediated delivery of Ca^2+^ from ER stores to mitochondria (De Vos et al., 2012; Stoica et al., 2014; Stoica et al., 2016; Gomez-Suaga et al., 2017; Paillusson et al., 2017; Gomez-Suaga et al., 2022). To determine how deletion of the PTPIP51 FFAT motif or coiled-coil domain might affect this function, we monitored mitochondrial and cytosolic Ca^2+^ levels in neuronal SH-SY5Y cells after IP3 receptor stimulation. For these studies, we stimulated IP3 receptor Ca^2+^ release by treatment with the muscarinic acetylcholine receptor agonist oxotremorine-M; SH-SY5Y cells but not HEK293 cells express this receptor. This approach, involving oxotremorine-M, has been used in many previous studies that examined the role of the VAPB-PTPIP51 tethers on ER-mitochondria Ca^2+^ exchange and so represents a particularly valid experimental route. SH-SY5Y cells have likewise been used in these studies (De Vos et al., 2012; Stoica et al., 2014; Stoica et al., 2016; Gomez-Suaga et al., 2017; Paillusson et al., 2017; Gomez-Suaga et al., 2022). Damage to the VAPB-PTPIP51 tethers is also seen in neurodegenerative diseases and so the use of neuronal SH-SY5Y cells is relevant in this context (Stoica et al., 2014; Stoica et al., 2016; Paillusson et al., 2017; Gomez-Suaga et al., 2022).

**FIGURE 4:**
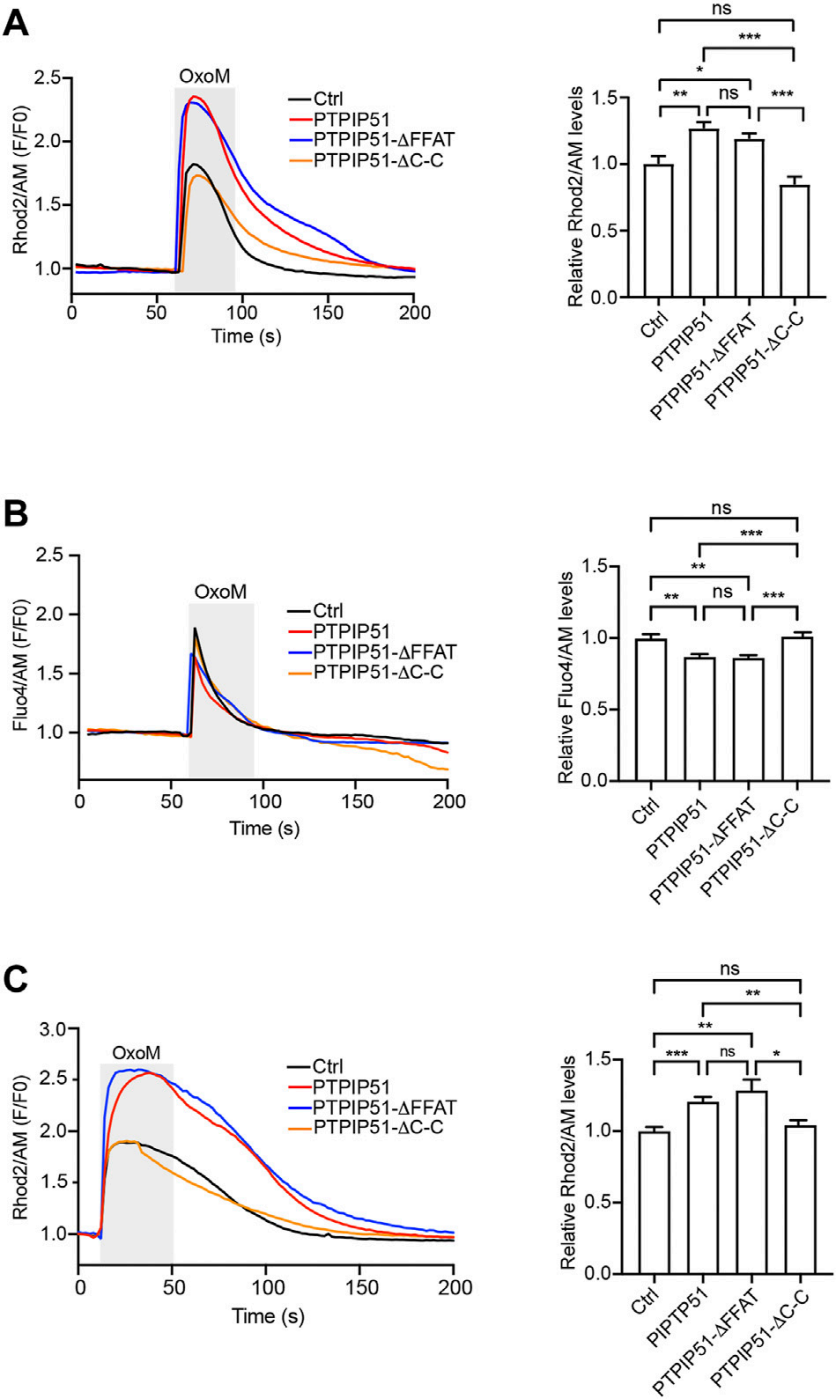
Deletion of the PTPIP51 coiled-coil domain but not FFAT motif disrupts IP3 receptor mediated delivery of Ca^2+^ to mitochondria. SH-SY5Y cells were transfected with EGFP **(A,C)** or DsRed **(B)** plus either control vector (Ctrl), PTPIP51-HA, PTPIP51-ΔFFAT-HA or PTPIP51-ΔC-C-HA. The release of Ca^2+^ was induced by treatment of cells with oxotremorine-M (OxoM). Mitochondrial **(A,C)** and cytosolic **(B)** Ca^2+^ levels weredetected using Rhod2/AM and Fluo4/AM respectively. **(A)** Representative Rhod2/AM fluorescence traces in the presence of external Ca^2+^ are shown on the left with OxoM treatment (depicted by the shaded area); normalised peak values are shown in the bar chart on the right. Expression of PTPIP51-HA or PTPIP51-ΔFFAT-HA induced similar significant increases in peak mitochondrial Ca^2+^ levels compared to control. Expression of PTPIP51-ΔC-C-HA had no significant effect on mitochondrial Ca^2+^ levels compared to control. Data were analysed by Welch ANOVA and Games-Howell’s post hoc test. N = 52–67 cells from three independent experiments. Error bars are s. e.m, ns, not significant; *p ≤ 0.05, **p ≤ 0.01, ***p ≤ 0.001. **(B)** Representative Fluo4/AM fluorescence traces are shown on the left with OxoM treatment (depicted by the shaded area); normalised peak values are shown in the bar chart on the right. Expression of PTPIP51-HA or PTPIP51-ΔFFAT-HA induced similar significant decreases in peak cytosolic Ca^2+^ levels compared to control. Expression of PTPIP51-ΔC-C-HA had no significant effect on cytosolic Ca^2+^ levels compared to control. Data were analysed by Welch ANOVA and Games-Howell’s post hoc test. N = 27–34 cells from three independent experiments. Error bars are s. e.m, ns, not significant; **p ≤ 0.01, ***p ≤ 0.001. **(C)** Representative Rhod2/AM fluorescence traces in the absence of external Ca^2+^ are shown on the left with OxoM treatment (depicted by the shaded area); normalised peak values are shown in the bar chart on the right. Expression of PTPIP51-HA or PTPIP51-ΔFFAT-HA induced similar significant increases in peak mitochondrial Ca^2+^ levels compared to control. Expression of PTPIP51-ΔC-C-HA had no significant effect on mitochondrial Ca^2+^ levels compared to control. Data were analysed by Welch ANOVA and Games-Howell’s post hoc test. N = 23–51 cells from three independent experiments. Error bars are s. e.m, ns, not significant; *p ≤ 0.05, **p ≤ 0.01, ***p ≤ 0.001.

We monitored mitochondrial Ca^2+^ uptake in SH-SY5Y cells expressing EGFP control vector, or EGFP plus either wild-type PTPIP51 or the PTPIP51 FFAT or coiled-coil deletion mutants (PTPIP51-ΔFFAT and PTPIP51-ΔC-C). In agreement with prior studies, oxotremorine-M induced a time-dependent increase in mitochondrial Ca^2+^ levels (Figure 4A) (De Vos et al., 2012; Stoica et al., 2014; Stoica et al., 2016; Gomez-Suaga et al., 2017; Paillusson et al., 2017; Gomez-Suaga et al., 2022). However, compared to control cells, the peak values were significantly higher in cells expressing wild-type PTPIP51 which is in line with previous studies and the known role of PTPIP51 in ER-mitochondria tethering (Stoica et al., 2014; Gomez-Suaga et al., 2017). Expression of PTPIP51-ΔFFAT also increased mitochondrial Ca^2+^ uptake and this increase was not significantly different to wild-type PTPIP51 (Figure 4A). However, the expression of PTPIP51-ΔC-C had no significant effect on mitochondrial Ca^2+^ values compared to control cells (Figure 4A).

We next monitored cytosolic Ca^2+^ levels after IP3 receptor stimulation in SH-SY5Y cells expressing DsRed control vector, or DsRed plus either wild-type PTPIP51 or the PTPIP51 FFAT or coiled-coil deletion mutants (PTPIP51-ΔFFAT and PTPIP51-ΔC-C). Again in agreement with prior studies, oxotremorine-M induced a time-dependent increase in cytosolic Ca^2+^ levels (Figure 4B) (De Vos et al., 2012; Stoica et al., 2014; Stoica et al., 2016; Gomez-Suaga et al., 2017; Paillusson et al., 2017). However, compared to control cells the peak values were significantly lower in cells expressing wild-type PTPIP51 (Figure 4B). This finding is in agreement with previous studies and is a consequence of the increase in ER-mitochondria tethering induced by PTPIP51 expression that enables rapid uptake of Ca^2+^ by mitochondria with a corresponding decrease in cytosolic Ca^2+^ levels (Stoica et al., 2014; Gomez-Suaga et al., 2017). Expression of PTPIP51-ΔFFAT also decreased peak cytosolic Ca^2+^ levels compared to control and this decrease was not significantly different to wild-type PTPIP51 (Figure 4B). However, the expression of PTPIP51-ΔC-C had no significant effect on peak cytosolic Ca^2+^ values compared to control cells (Figure 4B).

Finally, we monitored mitochondrial Ca^2+^ levels in the different transfected cells in media lacking Ca^2+^. These experiments produced similar results to those generated in the presence of external Ca^2+^. Thus, compared to control cells, the peak values were significantly higher in cells expressing wild-type PTPIP51 and PTPIP51-ΔFFAT but the expression of PTPIP51-ΔC-C had no significant effect on mitochondrial Ca^2+^ values compared to control cells (Figure 4C).

Thus, deletion of the PTPIP51 coiled-coil domain but not the FFAT motif abrogates the known stimulatory effect of PTPIP51 expression on IP3 receptor mediated ER-mitochondria Ca^2+^ exchange involving increased mitochondrial and decreased cytosolic Ca^2+^ levels (Stoica et al., 2014; Gomez-Suaga et al., 2017). These findings complement those described above on the roles of these domains in the binding of PTPIP51 to VAPB and ER-mitochondria contacts.

## Discussion

VAPB and PTPIP51 are ER-mitochondria tethering proteins that are of increasing scientific interest. This is because of their roles in several key cellular functions and because disruption to the VAPB-PTPIP51 interaction is seen in Alzheimer’s disease, Parkinson’s disease, and FTD/ALS (De Vos et al., 2012; Stoica et al., 2014; Galmes et al., 2016; Stoica et al., 2016; Gomez-Suaga et al., 2017; Paillusson et al., 2017; Gomez-Suaga et al., 2019; Lau et al., 2020; Yeo et al., 2021; Gomez-Suaga et al., 2022). Understanding the mechanisms that control the VAPB-PTPIP51 interaction thus represents an important area of research.

The present study used cellular assays to probe the roles of the PTPIP51 coiled-coil domain and FFAT motif in mediating the binding of PTPIP51 to VAPB, the formation of ER-mitochondria contacts, and IP3 receptor mediated delivery of Ca^2+^ to mitochondria, which are primary functions of the VAPB-PTPIP51 tethers. We found that deletion of the coiled-coil domain but not the FFAT motif disrupts all of these functions. Thus the PTPIP51 coiled-coil domain is an important mediator of VAPB binding and related functions. Our results also cast doubt on previous studies using *in vitro* peptide binding assays, which concluded that the interaction involves the PTPIP51 FFAT motif (Di Mattia et al., 2020; Yeo et al., 2021).

The reported K_d_ value for the interaction of the FFAT peptide with VAPB is high (52.87 μM), which represents a very low affinity of binding (Yeo et al., 2021). Thus, despite previous conclusions that the FFAT motif is key to binding PTPIP51 to VAPB, a thorough interrogation of the data obtained from these peptide binding studies provides results that are in general agreement with the cellular studies described here (Di Mattia et al., 2020; Yeo et al., 2021). Indeed, when describing their peptide binding K_d_ values, the authors state that the FFAT motif “only weakly contributes” to tethering (Yeo et al., 2021).

Phosphorylation of threonine-160 within the FFAT motif has been reported to be essential for PTPIP51 binding to VAPB, but these studies again involve *in vitro* binding of short peptides (Di Mattia et al., 2020). We are unaware of any data that demonstrates cellular phosphorylation of threonine-160 despite such analyses revealing 27 cellular/*in vivo* phosphorylation sites in human PTPIP51 (for a summary see https://www.phosphosite.org/proteinAction.action?id=984200&showAllSites=true). Since the VAPB-PTPIP51 interaction is readily detected in a variety of cellular assays including immunoprecipitation and proximity ligation assays (De Vos et al., 2012; Stoica et al., 2014; Stoica et al., 2016; Paillusson et al., 2017; Gomez-Suaga et al., 2019; Lau et al., 2020; Gomez-Suaga et al., 2022), it is of concern that no study to date has yet detected cellular phosphorylation of PTPIP51 threonine-160. Moreover, recombinant VAPB and PTPIP51 cytosolic domains produced in *E. coli* (i.e. non-phosphorylated) have been shown to interact robustly, which further queries the proposed essential role for threonine-160 phosphorylation in mediating the binding of PTPIP51 to VAPB (Stoica et al., 2014).

To summarise, this study showed that the PTPIP51 coiled-coil domain plays a role in mediating binding to VAPB, and the formation of ER-mitochondria contacts and linked IP3 receptor delivery of Ca^2+^ to mitochondria. Our findings also highlight the need for studies other than peptide binding when determining the structural determinants of protein-protein interactions. They do not eliminate the role of the FFAT motif in binding to VAPB but rather highlight that further work is needed in this area. Notably, more formal structural studies of VAPB binding to PTPIP51 are required. Considering the role of the VAPB-PTPIP51 tethers in several key cellular functions and that disruption of the interaction is seen in major neurodegenerative diseases, such work should be a priority.

## Data Availability

The raw data supporting the conclusion of this article will be made available by the authors, without undue reservation.
